# Intersections of power: videoconferenced debriefing of a rural interprofessional simulation team by an urban interprofessional debriefing team

**DOI:** 10.1007/s40037-021-00669-6

**Published:** 2021-06-09

**Authors:** Kathleen Dalinghaus, Glenn Regehr, Laura Nimmon

**Affiliations:** 1 Whitehorse General Hospital, Yukon Territory, Whitehorse, Canada; 2 Centre for Health Education Scholarship, Faculty of Medicine, University of British Columbia, Vancouver, BC Canada; 3 Centre for Health Education Scholarship, Department of Occupational Science and Occupational Therapy, Faculty of Medicine, University of British Columbia, Vancouver, BC Canada

**Keywords:** Critical theory, Sociomateriality (SM), Simulation-based education (SBE), Videoconferenced nurse/doctor dyad debriefing, Continuing professional development

## Abstract

**Introduction:**

Simulation as an educational tool is known to have benefits. Moreover, the use of simulation in continuing interprofessional development is vital in rural and remote communities with limited case volumes and resources. This study explored power dynamics between rural simulation participants and urban expert co-debriefers during a simulated operating room crisis and debriefing. The aim is to gain a rich understanding of rural/urban relational dynamics embedded within the constraints and affordances of videoconferencing technology.

**Methods:**

In situ observations of a videoconference-enabled simulation and debriefing were conducted, followed by seven semi-structured interviews, in this qualitative case study. A sociomateriality lens with additional sensitizing concepts of power from critical theory was employed to explore human and nonhuman interactions between rural learners, urban co-debriefers, and videoconferencing technology.

**Results:**

The interviews exposed subtle expressions of power dynamics at play that were curiously not observable in the enactment of the exercise. Rural learners appreciated the objectivity of the urban debriefers as well as the nurse/physician dyad. However, rural participants appeared to quietly dismiss feedback when it was incongruent with their context. Videoconference technology added both benefits and constraints to these relational dynamics.

**Discussion:**

Awareness of power relationships, and insights into affordances and constraints of videoconferencing may enhance operationalization of interprofessional simulation-based education (SBE) in rural and remote contexts.

## Introduction

Simulation-based education (SBE) provides learners with opportunities to practice rare and critical events in a safe setting. It can contribute to learning, maintenance of competence, continued professional development, and enhanced team performance [1–3]. A systematic review of SBE for health professionals described simulation as a positive experience with some evidence of immediate and long-term changes in knowledge, skills, and behavior [4].

Debriefing plays an integral role in SBE [5–7]. Self-debriefing options have been explored with some success [8]. However, the literature has raised doubts about our ability to accurately self-guide learning [9]. Thus, SBE best practices have emphasized the importance of guided debriefing with trained debriefers [10]. Importantly, learner physicians and nurses perceive feedback as more useful when it comes from their own professional group [11]. Thus, to optimize learning in interprofessional team-based simulations, the debriefing process may require multi professional co-debriefing dyads to ensure an understanding of the professions represented. This may add another layer of challenges, as co-debriefer dynamics “could be intensified in interprofessional SBE, where issues of status, hierarchy, and profession-related assumptions among debriefers and learners are in play” [10, p. 74].

SBE is especially appropriate in rural and remote communities, characterized by low case volumes and therefore fewer opportunities to utilize skill sets. In resource limited contexts, expert facilitators in debriefing are often not on site and must be sourced from urban contexts. Thus, in-person expert debriefing in rural contexts could entail a significant learner and/or institutional cost to contract expert urban debriefers (especially if multi professional co-debriefers are used). There is, as well, a personal cost in time and travel for the urban debriefers themselves. Real-time videoconferenced simulation and debriefing has been trialed as a possible solution to the human resource challenges of remote settings [12–14].

Although this might address the cost and accessibility issues, relational issues may also exist. For example, rural learners face the conundrum of being critiqued by a well-meaning ‘outside’ expert who may have no situated knowledge of their rural context [15], and may perceive the rural physicians’ context from a ‘deficit’ perspective [16]. Research into the use of videoconferenced simulation debriefings for remote participants is growing, but we still have little knowledge about the relational interactions in SBE between rural participants and urban experts. In particular, we know very little about how relational complexities, imbued with professional and geographic issues of power, might be exacerbated utilizing a videoconferenced interface.

In this study, our research question was “How do relational dynamics shape a rural team-based simulation with debriefing conducted by urban co-debriefing experts via videoconferencing technology?” Using an expanded sociomateriality lens [17, 18] with some sensitizing concepts of power from critical theory [19], our exploration of these dynamics was sensitized to the power dynamics between: interprofessional simulation participants, interprofessional co-debriefers, and the rural participants and urban debriefers. Our aim was to utilize these insights to guide SBE design and implementation in remote locations, and advance SBE research, informed by a deeper understanding of relationship complexities and the influences of videoconferencing technology.

## Methods

We conducted a single, qualitative, exploratory case study. Yin suggests this type of case study is suitable to “explore those situations in which the intervention being evaluated has no clear, single set of outcomes” [20, p. 548]. The University of British Columbia’s Behavioral Ethics Board (no. H18–03014) and the community hospital ethics committee granted ethics approval prior to data collection.

### Theoretical framework

Sociomaterial approaches have been utilized to explore both interprofessional learning [21] and SBE [22]. In particular, sociomaterial notions of ‘assemblages’ or ‘webs’ [18] resonate well with the dynamic, unique, emergent, and unpredictable nature of both human and nonhuman relational interactions during an interprofessional SBE event. In our analysis, we adopted a sociomaterial lens that was sensitive to how all entities have intrinsic or potential qualities that impact and shape assemblages, and how relational assemblages create actualized power or agency [17, 18]. To supplement this sociomaterial lens, we also utilized broad concepts from critical theory [19] as sensitizing concepts in our analysis to explore the enactment of power as it unfolds in context. This combination of sociomaterial and critical theory lenses allowed us to deepen our understanding of power relationships in the context of SBE video co-debriefing of rural practitioners by urban experts.

### Context

The simulation was performed in a northern Canadian community hospital operating room with local operating room staff. The hospital is situated over 2000 km by road or three hours by medevac flight from major tertiary hospitals. The operating room staff is composed of eleven operating room nurses, four general surgeons, two obstetrician gynecologists, one orthopedic surgeon, and eight anesthesiologists. At the time of the study an established simulation program did not exist at this community hospital.

### Participants

The principal investigator (KD) recruited local rural simulation participants to volunteer from the interprofessional surgical operating team through word of mouth, email and posters in the OR lounge. She informed potential participants that this simulation included a research component and provided a consent form that described the study but did not describe the particular simulation case scenario. One specialist surgeon, three general practice anesthesiologists and two operating room nurses volunteered for the event. However, the surgeon withdrew on the day of the simulation exercise, describing a scheduling conflict. Therefore, one anesthesiologist offered to play the role of the surgeon in the simulation.

At the local rural site, the simulation facilitator was a clinical nurse educator with some training in simulation technology. Another clinical nurse educator was recruited as an additional observer of the simulation, and to record notes. Both nurses were employed by the local hospital and offered to volunteer their time outside their regular work hours.

To access knowledgeable debriefers from an urban site, KD recruited one nurse and one physician debriefer by email from a contact list provided by an urban simulation program coordinator. The debriefers had never debriefed by means of videoconference before. These two individuals were from different tertiary hospitals and were not co-located. They had not previously co-debriefed together, but each had more than a decade of simulation and debriefing experience, and they were well acquainted with the Promoting Excellence and Reflective Learning in Simulation debriefing framework.

### Simulation description

One of the expert debriefers suggested the simulation exercise, a pediatric case of local anesthetic toxicity syndrome, because of previous experience with the scenario. This scenario was realistic and relevant for the rural context, as general surgeons administer local anesthetic blocks for such cases.

The simulation was conducted in situ in the operating room utilizing a Laerdal junior mannequin, its controller and vital sign monitor. The expert debriefer who had experience with the case was able to provide a simulation template that allowed the local rural clinical nurse educator to run the mannequin/monitors. The prebrief and simulation exercise was conducted for 13 min followed by 26 min for the debriefing component. The research team did not have any input into the debriefing strategies selected by the debriefers.

The debriefers connected to the simulation site via “Google Hangouts” on KD’s laptop computer using her personal hotspot to avoid hospital Wi-Fi interruptions. A high-definition endoscopy monitor was also connected to KD’s computer and the output was shared with the debriefers in real time. An additional camera was mounted on the endoscopy monitor to expand the debriefers’ field of view.

### Data collection

KD and the invited local clinical nurse educators with simulation experience observed and took field notes of the learners enacting the simulation, and of the post-simulation videoconference debriefing. A comparison of field notes and observations from the three research observers was performed the day following the simulation. This exercise provided the opportunity for divergent perspectives to be explored and referenced.

Within two weeks following the simulation scenario KD individually interviewed rural simulation participants in person, and urban co-debriefers over the telephone. These semi-structured interviews were audio recorded, transcribed, and de-identified prior to analysis.

### Data analysis

KD initially read and coded the transcribed interviews and field notes. Subsequently, the full research team KD, GR and LN met regularly to interpret and organize the codes in relation to the research question. GR and LN had no connection to participants and were able to provide their unique perspective to complement and counterbalance the insider interpretations of KD. The team used an inductive iterative approach to coding. Initially, anything that appeared related to the research question was coded as items based on participants’ descriptions of their experiences. As analysis progressed, we employed sensitizing concepts from sociomateriality theory [17, 18] and critical theory [19] inductively to develop patterns relevant to the research question. Analysis ceased when the research team became confident that there were no leaps of logic with respect to how our theoretically informed patterns related to the research question [23]. All coded data were organized and managed using Atlas.ti software.

At the time of the study KD was a practicing anesthesiologist with active hospital privileges at the rural community hospital site where this study was conducted. Thus, KD knew all the rural participants on a personal level. However, she had no role in promotion or disciplinary action with any of the participants and was unaware of prior relationship conflicts that would affect participation, data collection, or analysis. She did not personally know the urban co-debriefers. To ensure reflexivity, she documented her assumptions in a journal prior and subsequent to data collection as a way to illuminate her biases throughout the study. She also documented ‘ethically important moments’ such as emotionally vulnerable instances [24].

## Results

The participants generally felt that everyone learned something from the simulation experience. Within this overall seemingly positive educational context, three main research findings interlaced to provide insights into the relational dynamics that unfolded during a simulated crisis in the operating room: (1) the nature of interprofessional relational dynamics, (2) the impact of geography on relational dynamics, and (3) the ways videoconference (VC) technology mediated relational dynamics.

### The nature of interprofessional relational dynamics

In the simulation itself, stereotypical interprofessional power hierarchies did not appear to be salient. Both urban debriefers commented on how well the rural team worked together, “just like a well-oiled machine” (KD field notes). Interestingly, the urban debriefers, who did not know at the time of the simulation that the surgeon’s role was played by an anesthesiologist, both commented during the interviews that this might have played a role in the positive team dynamics observed in the simulation. For example, one debriefer noted that:

*they were very receptive to the anesthesiologist. And they had some suggestions, and I was like, “Wow, this is a surgeon that I’ve never known to act this way before. They’ve got amazing surgeons up there.”* (Urban Debriefer [UD] 2)

In subsequent interviews, the rural participants elaborated on the advantage of belonging to a small, stable group that has worked together for several years. The participants reflected that longstanding relationships in the group may have influenced the positive dynamics in the simulation. Participants noted that they view their coworkers as part of an extended family, as highlighted by the following comment by one of the participants:

*I mean that’s a big plus for our environment, and it speaks volumes about our team here. Most of us have been working *[together]* for five to ten years. So, there’s some family, there’s a comfort level. I think we have a mutual respect. *(Rural Participant [RP] 5)

For the rural participants, it appeared that interpersonal power dynamics rather than interprofessional power dynamics were more prominent in their reflections of the experience. Several acknowledged that participating in a simulation exercise invoked a level of performance anxiety that was exacerbated by the closeness of the team. Performance anxiety did not appear to be an explicit issue of interprofessional hierarchical power dynamics, as both nursing and physician groups expressed similar forms of vulnerability.

*No one wants to make a mistake, no one wants to look like they don’t know something in front of their colleagues. Because part of it too is then when we go into a real situation you rely heavily on everyone around you and I think people maybe might be afraid of appearing like unknowledgeable in front of their colleagues and then losing that trust in an emergency.* (RP 4)

### The impact of geography on relational dynamics

The urban/rural duality between debriefers and participants appeared to foster both positive and problematic relational dynamics in terms of the ways that participants interfaced.

Although rural learners recognized the ‘outsider’ status of the urban debriefers, they appeared to appreciate the debriefers’ content knowledge and debriefing expertise. In general, they found the urban perspective credible and informative as expressed by one participant: *“I certainly think they might have a fresher, more up to date, at their fingertips way of thinking about those things *[simulation crises]*” *(RP 3).

Moreover, the rural participants valued the potential for objectivity in urban debriefers’ assessments because they did not share history or politics with the participants (suggesting again, possible hidden interpersonal relational power dynamics between rural participants). Therefore, the ‘outside’ critique was viewed as potentially providing psychological safety and objectivity as exemplified by this participant’s comment:

*I like having … somebody else debrief, like not from our site because it leaves them—you don’t have to be wary—as wary. I mean everybody’s wary of peoples’ feelings, but you don’t have to dance around personal dynamics and interprofessional dynamics and you can give feedback honestly. They don’t have any history. There’s no politics involved. *(RP 1)

Despite the receptivity to the ‘outsider’ perspective, however, the urban feedback was filtered by rural participants through a situated contextual lens. Rural participants did not neutrally accept the feedback if they perceived it as not reflecting the reality of their context. This was especially salient with respect to time and available resources. For example, a criticism from one debriefer was that it took nine minutes after the announcement of the diagnosis before intralipid was given (KD’s field notes). In a subsequent interview, one rural participant situated this comment in the reality of the rural context and countered:

*I think if you are urban and you’ve never practiced rural, there is a fundamental lack of understanding of what the skill levels look like. The difference I think between us and them is our resources and the skill level of our resources, and especially at night what that would look like even in a well-practiced group, with our resources I’m not sure that the expectation of time to intralipid was realistic. *(RP 1)

Interestingly, this urban/rural difference was not raised during the simulation debriefing itself by rural participants. Therefore, an opportunity was lost for rural participants to negotiate a possible co-constructed resolution with urban debriefers.

### The ways videoconference (VC) technology mediated relational dynamics

The videoconference (VC) link mediated human activity in ways that appeared to foster dualities in terms of producing both enabling and constraining effects. The VC link offered the ability to bring outside expertise to a rural site, provided significant time savings, and minimized debriefers’ time away from their other professional obligations. Co-debriefers did not need to travel to the rural site, and could take part in debriefing at a location of their choice: *“I was on my couch in my living room” *(UD 1).

There was a clear recognition of the value of VC technology to enable multi professional representation in the debriefers, as noted by one participant: *“I think it was a good perspective to have someone … be the expert and sort of focus on the physician role and then have someone be the expert and focus on the nursing role”* (RP 4).

Moreover, the urban presence via the VC link was perceived by simulation participants as being unobtrusive. All the rural participants commented that the VC link enabled the urban debriefers to effectively ‘disappear’ as noted by this participant’s comment: *“I remember thinking that there were people observing, but I sort of forgot about the video aspect of things” *(RP 2).

However, the VC link added complexity to co-debriefing in a number of unique ways. Debriefers did not have the opportunity to meet as usual immediately after the simulation scenario to discuss what had transpired in the simulation, to ‘get on the same page’, and plan a debriefing approach. *“Who to take the lead on the debrief … so that’s the one drawback to the—like that live video feed SIM, is that the debriefers can’t set up their roles ahead of time”* (UD 2).

Debriefers also appeared to have difficulties assessing the rural participants’ nonverbal emotions during the simulation and debriefing. Valuable nonverbal cues to guide and enrich the debriefing were difficult to capture as exemplified by the following quote: *“I feed a lot on how people are feeling and kind of that reactions piece. So, I felt like being over video I kind of lost that. You have to rely more so on what people are saying” *(UP 1).

## Discussion

Interprofessional power hierarchies are well documented in the health professions literature [25, 26]. Although there were relatively few overt interprofessional issues in the enactment of the simulation itself, innuendos and remarks by the rural participants during the interviews also suggested that there were politics and history woven into the interpersonal, and interprofessional relational dynamics amongst the rural team that may have been at play. These longstanding relational dynamics could potentially have affected the learning. Our use of Fenwick’s [17] and Müller’s [18] expanded views of sociomateriality sensitized us to these subtle interprofessional power dynamics as they related to the simulation experience. For example, Müller [18] suggests that affect and emotion are sociomaterial and influence how assemblages hold together or fall apart. Indeed, emotional vulnerability, both personally and professionally, were described by both the nurse and physician rural participants. Research has suggested that full engagement in interprofessional learning might be limited by threats to both personal [27] and professional [25, 26] power. In this regard, the fact that videoconferencing technology enabled two urban debriefers from different professions to co-debrief seemed to have been particularly valuable. There was a strong sense from participants that the use of ‘outside’ debriefers offered an important mechanism to finesse these longstanding relational dynamics and allow for more authentic (objective) discussion of the team’s performance. This perception of outside objectivity may have been emphasized due to the urban co-debriefing dyad that was composed of a nurse and physician. In fact, Cheng and colleagues [10] have highlighted the value of interprofessional co-debriefing bringing diverse perspectives, and this may well have contributed to a sense of ‘fairness’ in the feedback being offered in this context.

The urban/rural dynamic was exemplified by the manner in which participants brought their rural contextual lens to the input provided by the urban debriefers. This filtering of information based on the rural participants’ local context appeared not to be a sign of disrespect, but rather indicative of their local reality, especially in consideration of available resources. However, the absence of responses from rural participants to contextually incongruent input from urban debriefers during the debriefing itself was intriguing. It may suggest that the rural participants did not feel empowered or sufficiently connected to the debriefers to engage with them. For example, the rural participants might have offered insight into local resources, social norms, and policies in ways that might have led to rich discussions regarding how to manage these issues. However, rather than the discrepancies in perspective being effectively addressed and situated in the rural context by rural participants, the debriefers’ feedback appeared to be quietly dismissed. This echoes an aspect of Telio and colleagues’ [28] research on the complex credibility judgments made by learners that impact feedback uptake based on learners’ perceived sense of being understood and valued in the eyes of the debriefers. Furthermore, Bourke and colleagues [29] challenge the stereotyped ‘deficit’ view of rural and remote settings, and suggest an expanded, balanced perspective that captures the strengths and dynamism of rural practice settings. We propose that if debriefers are not intimately familiar with the context of the simulation participants, they must be careful to ensure that participants feel sufficiently empowered to share their own situated knowledge of the event. Otherwise, there is risk of losing the ability to authentically co-construct ways to effectively address those local concerns.

Finally, our interest in the assemblage of the social and the material highlighted the need to recognize that the videoconferencing modality is not ‘neutral’. Rather this modality was actively contributing to and shaping the assemblages, and the hues of power within the intermingled professional, geographic, and technologic relational webs in this complex simulation debriefing context [17, 18]. For example, the unobtrusive nature of the video observations allowed participants to become more fully engaged in the simulation than they might otherwise have been. However, there were several ways in which the video presence of interprofessional urban debriefers who were not co-located might have exacerbated some relational issues. Indeed, the flow of events was altered by the video connection in that debriefers did not feel able to connect with each other immediately following the simulation. In retrospect, it is not that such a meeting could not have happened. However, it appears that in the context of an already established three-way video link between the debriefers and the simulation site, this was simply not considered until too late. Further, because of the technology, the debriefers felt somewhat limited in their ability to read each other’s, and the rural participants’ emotive non-verbal cues. This likely led to additional complexities in their ability to establish leadership and followership roles in the debriefing, to coordinate with each other, and to connect with participants to foster a smooth discussion during the debriefing, all of which are identified as important components of effective co-debriefing processes [10]. We also note that the video link placed inadvertent constraints on ‘interaction’ time. The event was planned for a certain time and the video link was established to coincide with the start of the event. There was no ‘casual’ engagement between the rural participants and the urban debriefers as might have occurred if everyone was physically present. This may well have limited the ability of the debriefers to develop rapport or to get a sense of the rural context. Again, we can only speculate, but we do wonder about the extent to which the rural dismissal of some of the urban feedback might have been exacerbated by this lack of socializing opportunities that the videoconferencing medium naturally imposed.

Our findings and interpretations are necessarily limited by the study design. This study involved a single research site with a limited sample size. The small number of participants was appropriate for this study that was theoretically informed, had quality dialogue/data, and involved in-depth analysis that richly answered our research question [30, 31]. However, it remains open for others to consider whether these insights resonate with their context in terms of relevance to the design of future video-based debriefing strategies for rural simulation. Additionally, volunteer bias may have influenced the data by not being representative of the general characteristics of the OR team. Finally, because technology is continuously evolving, this study only captures a specific technological intervention in a moment of time.

These limitations notwithstanding, our findings have potentially important implications for the use of simulation to support learning in geographically isolated settings. Thoughtful, contextually attuned design of SBE informed by our research results has the potential to maximize the value of SBE, including the motivation to engage in simulation learning and the uptake of feedback. This could involve planning different co-debriefing approaches and strategies depending on whether debriefers are co-located or not. A more thorough prior introduction of the rural context and rural resources may be critical. This localized orientation may improve the educational outcomes of the simulation as relationships and the materiality of SBE assemble and disassemble in emergent and unique ways.
